# Contribution to the Study of the Horse-chestnut

**Published:** 1897-02

**Authors:** 


CONTRIBUTION TO THE STUDY OF THE HORSE-CHESTNUT. The hog, which eats acorns, roots, and divers fruits so greedily, absolutely refuses to touch the horse-chestnut. The recommendation not to give him this fruit in large quantities is therefore wholly useless. The duck likewise refuses them under whatever form they are presented. Mr. Comevin found that fresh, decorticated horse-chestnut in doses of 40 to 50 grains per day poisons the duck. Although not exempt from the effects of the poison, the chicken offers a greater resistance than the duck, and the pheasant a still greater one than the hen. Neither desiccation nor torrefaction destroys the toxicity of the horse-chestnut. Maceration and especially boiling in a large quantity of water remove the poison. A mixture of finely pulverized horse-chestnut and beets or other fleshy roots, when ground very fine, is readily eaten by sheep, which can consume from one to two kilogrammes of fresh horse-chestnut daily without injury to their health. This immunity is shared by all the ruminants, domestic and wild. The poisonous principle of the horse-chestnut cannot be tannin nor the oil which it contains, but a sort of soapy substance identified by M. Fréry with saponin. This poison is very active in the duck, which it kills rapidly. It produces effects intermediary between those of the saponin of mildew and the extracts of the autumnal meadow saffron.—*Annales de Méd. Vét.*, March, 1896.

